# A Pilot Study on Behavioural and Physiological Indicators of Emotions in Donkeys

**DOI:** 10.3390/ani13091466

**Published:** 2023-04-25

**Authors:** Samanta Seganfreddo, Diletta Fornasiero, Marta De Santis, Franco Mutinelli, Simona Normando, Laura Contalbrigo

**Affiliations:** 1 National Reference Centre for Animal Assisted Interventions, Istituto Zooprofilattico Sperimentale delle Venezie, Viale dell’Università 10, 35020 Legnaro, Italy; 2 Epidemiology and Risk Analysis in Public Health, Istituto Zooprofilattico Sperimentale delle Venezie, Viale dell’Università 10, 35020 Legnaro, Italy; 3 Department of Comparative Biomedicine and Food Science, Università degli Studi di Padova, Viale dell’Università 14, 35020 Legnaro, Italy

**Keywords:** donkey, animal emotion, welfare indicator, animal behaviour, behavioural diversity indexes, operant conditioning

## Abstract

**Simple Summary:**

Animal welfare science is facing the challenge of measuring the subjective feelings and emotions of animals. In the case of donkeys, this could lead to a better understanding of their specific needs, improved training methods, and a healthier relationship with humans and the environment from a holistic perspective that considers the interconnection between the human, animal, and environmental health and welfare (One Health-One Welfare). In this study, we tried to overcome the difficulty of reading animal emotional states by means of some of the non-invasive parameters cited in the scientific literature. These behavioural and physiological parameters were used to compare the emotional response of donkeys in two situations of different emotional content in order to find possible indicators of positive or negative emotions in this species. The results showed significant differences in behavioural (in terms of ears orientation, postural changes, and behavioural complexity) and physiological (heart rate variability) responses when comparing a rewarding vs. a frustrating context. Despite the small sample considered for the analysis, which limits the possibility of generalization of the results, this preliminary investigation highlights promising outcomes and indicates that further investigation of these non-invasive parameters could lead to the identification of valid emotional state indicators in donkeys.

**Abstract:**

Recognizing animal emotions is critical to their welfare and can lead to a better relationship with humans and the environment, especially in a widespread species like the donkey, which is often prone to welfare issues. This study aims to assess the emotional response of donkeys through an operant conditioning task with two presumed different emotional contents. Specifically, a within-subject design including positive and negative conditions was conducted, collecting behavioural and physiological (heart rate variability and HRV) parameters. Facial expressions, postures, and movements were analysed by principal component analysis and behavioural diversity indexes (frequencies, activity budgets, richness, Shannon and Gini-Simpson). During the positive condition, both ears were held high and sideways (left: r = −0.793, *p* < 0.0001; right: r = −0.585, *p* = 0.011), while the ears were frontally erected (left: r = 0.924, *p* < 0.0001; right: r = 0.946, *p* < 0.0001) during the negative one. The latter was also associated with an increased tendency to walk (r = 0.709, *p* = 0.001), walk away (r = 0.578, *p* = 0.012), more frequent changes in the body position (V_Body position_ = 0, *p* = 0.022), and greater behavioural complexity (V_Gini-Simpson Index_ = 4, *p* = 0.027). As for HRV analysis, the root mean square of successive beat-to-beat differences (rMSSD) was significantly lower after the negative condition. These non-invasive parameters could be considered as possible indicators of donkeys’ emotional state.

## 1. Introduction

Nowadays, animal emotions are the focus of animal welfare science [1,2,3]. Briefly, emotions can be defined as innate response systems that are experienced as intense and short-lived affective states to a certain event [4]. They involve different components (i.e., subjective, physiological, behavioural, and cognitive), and each of these components presents a valence (i.e., pleasant or unpleasant), a degree of arousal, and a duration [3,5,6]. From the point of view of affective states, adequate animal welfare can be settled as the absence of long-term or steady negative emotions (or moods), combined with the opportunity to experience positive feelings [7,8].

Despite the growing interest in the emotional lives of animals [3], the measurement of affective components in animal beings presents some inherent difficulties [9,10,11], such as the absence of verbal communication [12] and the wide variety of behavioural patterns and emotional repertoires among species [9]. In particular, this becomes even more complicated when it comes to analysing positive emotions, the expression of which is commonly regarded as more subtle [7,13].

Donkeys play different roles in human society: they are used for work, production, and transport, but are also kept as pets or involved in animal-assisted interventions (AAI) [14,15]. A better understanding of their emotions could lead to a healthier relationship with humans, in which ‘positive welfare’ (see [16] for a review on this concept) is promoted [17], and possibly a virtuous circle is established to the benefit of the animal, humans, and the environment.

The implications of a better understanding of animal emotions are multiple. They relate to understanding our own psyche (with a comparative approach); to learning how to care more effectively for our livestock, working animals, and pets—which also has positive implications in practice and from a production perspective; finally, even to gaining insight into the health of an ecosystem (according to the One Health-One Welfare perspective) [5,18].

To date, a number of non-invasive measures have been used in an attempt to assess the affective states of animals [5]. Among these, behavioural measures such as facial expressions and body postures have shown the potential to reflect different emotional states [19,20]. In particular, the Grimace Scales were developed from the study of facial expressions. These scales originally emerged as a method to measure pain in human individuals unable to express themselves verbally [21] and were later developed for other species, starting with laboratory rodents (mice and rats) [21,22] and then sheep [23], cats [24], horses [25], and recently also donkeys [26]. Regarding donkeys, two other scales that consider facial expressions and body postures for pain assessment have recently been developed and tested [27,28], while other studies have considered donkeys’ behavioural responses to pain (nociceptive threshold testing) [29] and analgesic administration [30]. Apart from pain, facial expressions have also been proposed to investigate positive emotional states in animal species as well [7,19,20]. However, to our knowledge, no investigation has been conducted to study facial features and donkey postures as possible indicators of emotional response to positive and negative (but not painful) stimuli. Furthermore, behavioural diversity, a concept derived from ecology, recently has been proposed as a potential indicator of positive animal welfare [31]. Behavioural diversity reflects the level of complexity of the behavioural patterns exhibited by animals and is believed to be indicative of animal welfare, as animals with higher behavioural diversity are likely to be less restricted and, therefore, are able to display their natural behavioural repertoire more freely [31]. Despite the desirability of indicators of positive welfare in animals, to our knowledge, behavioural diversity indexes have not yet been analysed in donkeys or in a similar context to the present study.

Apart from the behavioural component, affective responses can also be detected by a series of physiological cues or biomarkers, since they involve a complex interplay between the autonomic nervous system, neuroendocrine system, and immunological activation. Among these cues, the analysis of heart rate variability (HRV) has been increasingly used as a research tool for the assessment of emotions and welfare in animals [5,7,32]. Specifically, HRV measures fluctuations in the time interval between successive heartbeats, which are regulated by the autonomic nervous system [32,33]. In donkeys, this parameter has been studied as an indicator of stress by two studies so far [34], one comparing HRV parameters with two different training methods [35], the other analysing HRV variation before, during, and after an AAI session [36]. In contrast, HRV analysis was performed more frequently in horses, where it was used to investigate their emotional state in response to different stimuli [37,38,39,40]. Given its premises as a possible welfare indicator and the little information actually available on donkeys, HRV analysis was chosen to be explored in the present study.

Therefore, this study aimed to assess the emotional response of domestic donkeys when confronted with two situations of presumed different emotional content (i.e., positive vs. negative). To achieve this objective, two different test conditions were designed and implemented, collecting both behavioural (facial expressions and postures) and physiological (HR and HRV) parameters.

## 2. Materials and Methods

### 2.1. Ethical Statement

The research protocol of this study was approved by the Ethics Committee of the Istituto Zooprofilattico Sperimentale delle Venezie (EC protocol number CE_IZSVe 8/2021) and was outside the scope of the Italian legislation on the protection of animals used for scientific purposes (D.L.vo n. 26 of 4 March 2014), which implements the corresponding Directive 2010/63/EU.

### 2.2. Animals and Farm Selection

For the purpose of this study, one farm of donkeys involved in AAI was selected. The selection criteria for the farm included: the number of animals, the presence of suitable areas for carrying out the training and tests, and the fact that animals were properly detained. Specifically, donkeys were group housed in two internal corrals with access to an external paddock. They were fed twice a day with limited hay and had pasture during the warm season, while water was available ad libitum. In the chosen facility, there were 19 asses of different ages, breeds, and gender (females n = 15; males n = 4). They were non-productive and non-working animals occasionally involved in human-animal interactions and AAI. All the donkeys were evaluated for eligibility to participate in the data collection, following the inclusion/exclusion criteria reported in Table 1. A total of 14 adult non-working and non-productive donkeys of both sexes were selected to be included in the study (females n = 11; males n = 3).

Among the chosen donkeys, 5 females were excluded at a later stage due to oestrous onset (n = 3), lack of motivation in performing the task (n = 1), and following an injury (n = 1). Information about donkeys’ age, sex, and height at the withers was collected along with the anamnesis of each individual. More details on animal selection and management are reported in a previously published study [41].

### 2.3. Experimental Design

A within-subject design including a positive and negative condition was carried out to investigate emotional expression in the two opposite contexts. Specifically, a first test with ‘positive’ reinforcement and a second test with a ‘negative’ stimulus were used to investigate the facial expressions and postures of donkeys respectively in an allegedly positive (i.e., rewarding) and negative (i.e., frustrating) context. To induce the positive emotional state (positive (+) test), a feeding reward was used by exploiting an operant conditioning mechanism. The same feed reward was used successfully as reinforcement during the training phase [41]. On the other hand, the failure to receive feed has been assumed to elicit a negative emotion in donkeys under study (negative (−) test), as has been done in analogous studies conducted, for example, in cows [42], in calves [43], and in sheep [44].

Both physiological data (e.g., heart rate variability) and behavioural responses were recorded during the tests.

To reach the study’s objectives, the selected animals were involved in operant conditioning as described by Seganfreddo et al. (2022) [41] (the process is also briefly summarized in the ‘Training and test’ section of the present work). The three researchers involved in the data collection were females, always with tied hair (i.e., ponytail), and dressed in blue jeans and blue pile fleece. Researcher A was the one in charge of taking the animal from the pen and leading it to the test area; researcher B had the task of remaining in the test area next to the donkey in order to promptly intervene, in case of need, during the test phase; and researcher C was in charge of quickly providing the animal with positive reinforcement/negative stimulus.

The positive reinforcement consisted of a palatable feed reward (i.e., 20 g of mixed wholemeal flakes) and the negative stimulus consisted of medium-sized stones (i.e., non-edible material). Hay was always available for the animals while they were being tested. It was placed on both sides of the test area, in the space where animals of both corrals are normally fed. This choice was based on the concept of avoiding additional stress for both tested and untested animals by over-modifying their habits and environment.

#### 2.3.1. Test Area and Manipulandum

**Figure 1:**
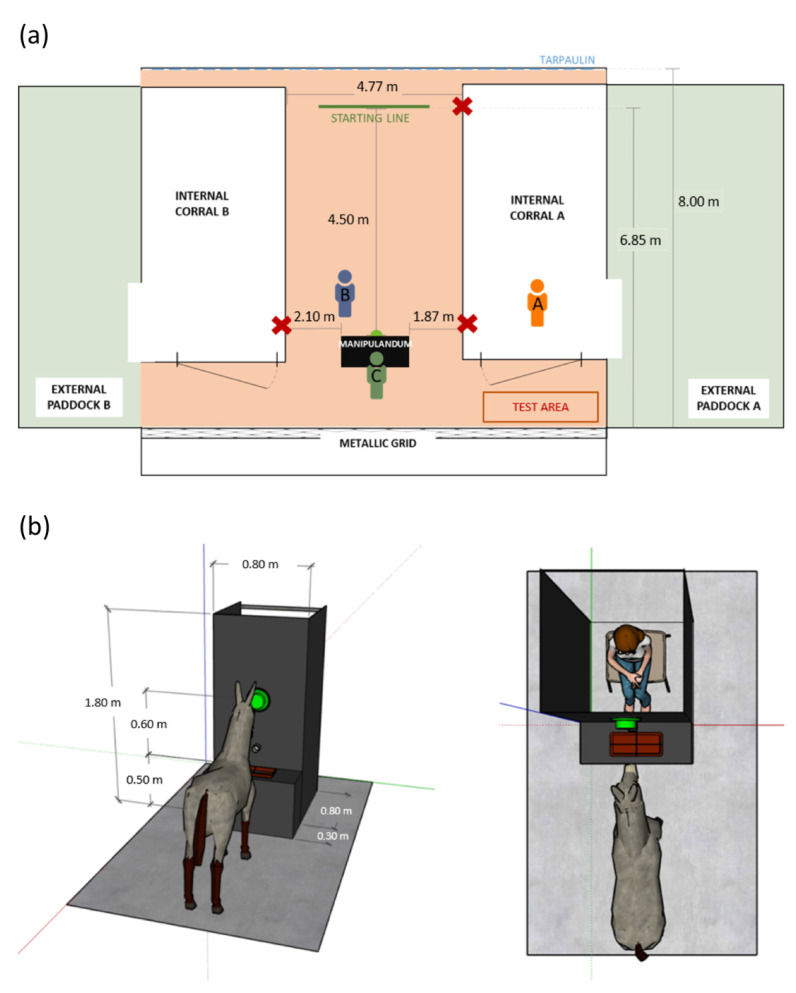
(**a**) Test area design. Red crosses: points where the cameras were placed; human shapes labeled with A, B, and C: researchers involved in the data collection; light red area: test area where the animal was allowed to move during the test sessions. (**b**) Left panel: frontal view of the manipulandum; right panel: view from above. The apparatus was composed of a cabin, a green button, a hose, and a tray. Following a complete buzzer pressure, the donkey could receive the reinforcement without being able to see researcher A, positioned inside the cabin. The drawing was made using SketchUp for Web.

The test area was structured as depicted in Figure 1a, with a manipulandum located at the end of the corridor. The manipulandum was an apparatus specifically designed and comprised a cabin with a green button on the front side (Figure 1b). Researcher C provided the positive reinforcement or the negative stimulus from inside the cabin as soon as the donkey pressed the button. In order to obtain the reinforcement, the pressure had to be ‘complete’, with the button lighting up and making a sound automatically. The ‘not complete’ pressures, i.e., the ones that did not make the buzzer light up and ring, were noted as ‘attempts’ of pressure and did not provide any reward to the animal.

#### 2.3.2. Training and Testing

The donkeys underwent the test phase following an initial acclimatization phase and an operant conditioning training period using the same manipulandum described above. Briefly, training consisted of 4 learning sessions with 15 maximum obtained rewards or approximately 10 min duration, and 1 final session of 10 min or a maximum of 10 rewards. The learning criterion was performing 10 successive button presses independently (i.e., without any intervention from the researcher). The detailed description of the training phase can be found in Seganfreddo et al. (2022) [41]. The positive reinforcement used during the positive (+) test was the same utilized during all the training phases, while the negative stimulus was utilized merely in the negative (−) test. During the training and the tests, the animals in the side fences were always allowed to observe the tested donkey placed in the test area. Water was not present in the test area where the animals stayed 15 min maximum for each test session. The positive and negative tests were conducted with the (+) test always being carried out before the (−) one. The (+) test ended after a maximum of 10 min or after the animal obtained 10 positive reinforcements. The (−) test ended after 10 min or after the donkey gained 5 negative stimuli, with two exceptions (i.e., two donkeys obtained 6 and 7 negative stimuli, respectively, as a result of a very rapid pushing of the button, before the subjects could be moved away from the test area). The limit of only 5 pressures in the negative test was set because the animals were showing signs of restlessness and to prevent them from damaging the manipulandum. Table 2 displays the different phases of training and testing.

### 2.4. Behavioural Observations

**Figure 2:**
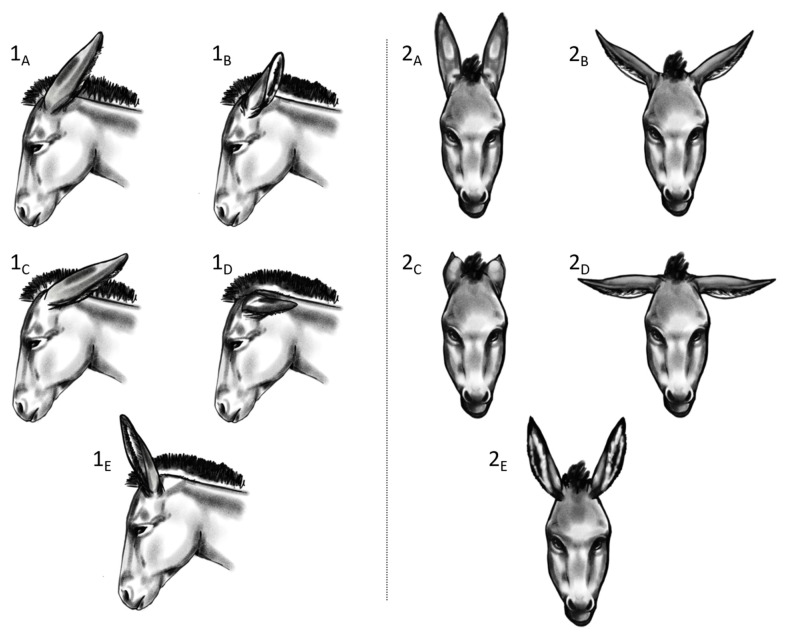
Donkeys’ ear position illustrative ethogram. (**Left panel—1**): lateral view; (**right panel—2**): frontal view. Ears positions: (**A**): back-up; (**B**): side-up; (**C**): back-down; (**D**): side-down; (**E**): front-up.

**Figure 3:**
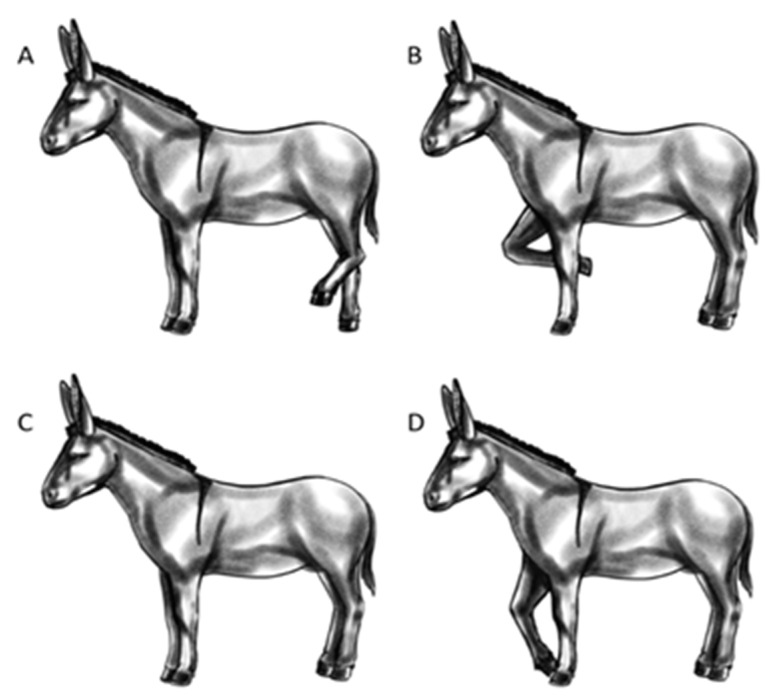
Standing position illustrative ethogram. (**A**): 3-legs standing; (**B**): bent knee position; (**C**): 4-legs standing; (**D**): pointing position.

The (+) and (−) test sessions were video-recorded by 3 cameras that were positioned in key spots (Figure 1a). A single video per donkey for each test was then created by synchronizing, via audio, the 3 video tapes using Movavi video suite software (Movavi, St Louis, MO, USA), to show simultaneously the three cameras’ perspectives. The video collages obtained of the (+) and (−) tests were analysed independently, using the BORIS software [45], by 2 not-blinded observers who recorded all the relevant behaviours manifested by donkeys. The behaviours that were considered for the present study and their definitions are reported in Table 3. In addition, the graphical representations of some animal postures (i.e., standing and ear positions) are presented in Figure 2 and Figure 3.

Inter-observer reliability was examined using Cohen’s Kappa (K) [46] by considering the number of recorded behaviours for each animal and each test-type by each of the two observers.

### 2.5. Heart Rate and Heart Rate Variability Analysis

To collect the cardiac beat-to-beat (R-R) interval series, all donkeys involved in the experiment were equipped with a Polar Equine Science belt (Polar V800 with H7 sensor), following the instructions provided by the manufacturer (Polar Electro Oy, Kempele, Finland). An ultrasound transmission gel (Cogel Ultrasound, Comedical s.r.l., Trento, Italy) was applied to the electrodes of the Polar belt to facilitate the signal transmission. The animals were accustomed to the application of the Polar heart rate monitor before the experimental phase. Subsequently, RR intervals were recorded in each donkey during the positive (+) and negative (−) tests, and for 10 min before (used as the baseline and indicated as ‘pre’) and after the tests (denotative of the return to homeostasis, and indicated as ‘post’), with donkeys free to move in their corral, in a resting condition. The collected data were uploaded to the Polar Flow software, provided by the manufacturer. Raw recorded R-R interval time series were downloaded from Polar Flow and then imported into Kubios software (Kubios HRV Standard 3.4.1) for the HRV analysis [47]. The frequency bands were set as those indicated by Kuwahara et al. (1996) [48] for the horse, given the fact that they have not yet been determined in the donkey, and that they belong to the same genus (*Equus*). No detrending was applied to these data, while artifact correction was set at 0.4 on the basis of a recent study that validated the use of Polar V800 in the horse [49]. Due to the poor quality of the signal, data on five donkeys were partially excluded from the analysis. For the remaining data, mean heart rate (HR), mean RR, and HRV time domain parameters relative to one-minute epochs were calculated. Specifically, the standard deviation of beat-to-beat intervals (Std RR, ms) and the root mean square of successive beat-to-beat differences (rMSSD, ms) were chosen as indicators of the sympathovagal balance [50,51]. Spearman correlation coefficients were calculated between HR and RR, and rMSSD and Std RR, respectively, in order to verify the inverse correlation that exists between HR and HRV, as described in the literature [52]. Differences in the HR and HRV parameters were then evaluated considering: (i) the comparison of data recorded during the pre-test-post study phases separately for the (+) test and the (−) test and, (ii) the comparison of data collected during the same phases for the two types of test (i.e., pre (+) test vs. pre (−) test, (+) test vs. (−) test, and post (+) test vs. post (−) test). Due to the presence of some missing records, the partially overlapping *t*-test for samples with paired and unpaired observations was chosen for this analysis.

### 2.6. Statistical Analyses

A preliminary descriptive analysis of the data was performed, together with some exploratory representations of the identified behaviours recorded for each animal, to identify the behavioural variables of greatest interest to investigate donkey emotionality. To allow the comparison between the behavioural patterns recorded during the two tests, the observation time of both sessions was limited to the shortest duration recorded for each subject.

Facial expressions, postures, and movements (calculated as the percentage duration of each behavioural manifestation for each subject) were analysed using principal component analysis (PCA). The latter is an exploratory technique that increases data interpretability, transforming high-dimension data into lower-dimension data while retaining as much information as possible [53]. The aim of the PCA was to provide an overview of potential facial expressions, postures, and movements significantly associated with each test group (i.e., positive and negative). The different facial expressions and postures were included in the analysis as behavioural variables of interest and the two test groups as illustrative variables to assess the association between the manifestation of certain behaviours in the context of the administration of a rewarding stimulus ((+) test) or, conversely, an unexpected and frustrating stimulus ((−) test). The Wilcoxon signed-rank test was applied to evaluate significant differences in the subjects’ scores obtained for the first three principal components (PCs), to determine the ones most associated with the subjects’ grouping defined by the test type. Once identified, the Spearman correlation coefficients were calculated between the significant PCs and the list of behaviours included in the analysis, to classify the groups of modes mostly manifested by the animals in the two different contexts.

Behavioural diversity was investigated in our study through a series of indices to evaluate potential differences in the range of behaviours exhibited during the two testing sessions, applying the non-parametric Wilcoxon signed-rank test. For the calculation of diversity indices, all the state events were considered with the exception of the ‘area exploration’, which was never observed in the study group, and the ‘stones exploration’, as it could occur only during the (−) test, according to the study design. The list of the behavioural diversity indices included in the analysis, their interpretation, and their possible values range are presented in Table 4.

All the data cleaning and preparation, graphics, and analysis were performed using R statistical software 4.2.2 [54] and R Studio [55] and the following packages: stats [54], vegan [56], and Partiallyoverlapping [57] for the statistical tests and analyses; ggplot2 [58], ggbiplot [59], and ggrepel [60] for the graphical representations of the results.

## 3. Results

### 3.1. Behavioural Observations

The inter-observer reliability in the behavioural observations ranged from K = 0.89 to K = 0.94 depending on the animal and test. All the state and point behaviours recorded during the two test sessions are graphically presented, for each subject, in the Supplementary Material Figure S1.

**Figure 4:**
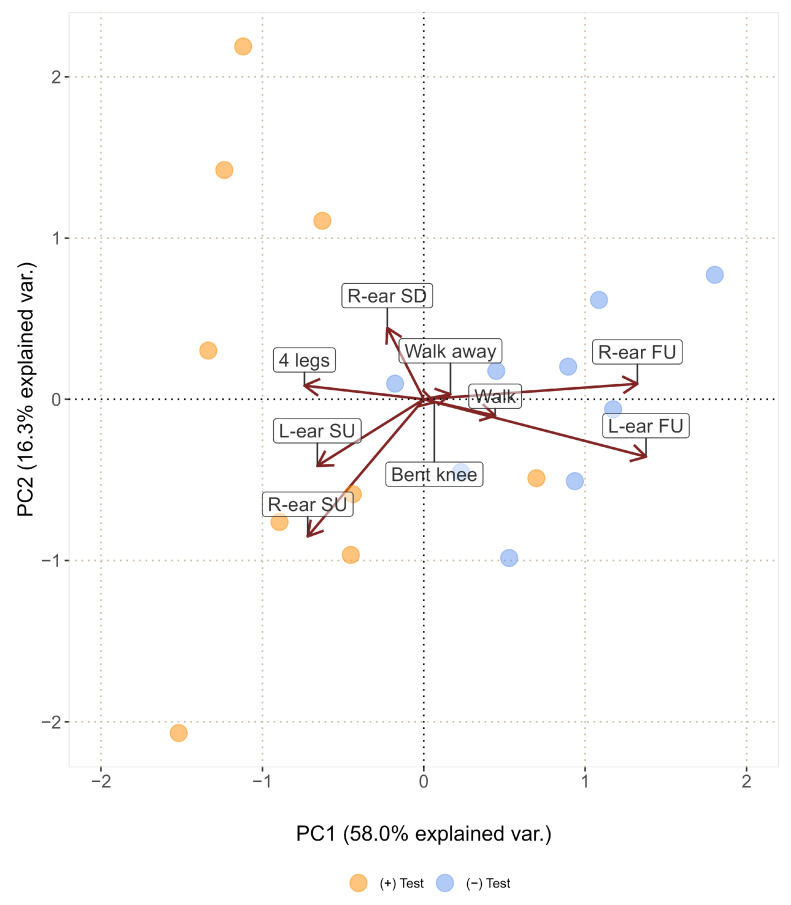
Principal component analysis. X-axis: PC1 score; y-axis: PC2 score; dots: subjects’ scores according to the two testing sessions; red arrows: vectors representing behaviours significantly associated with PC1.

The PCA identified the behaviours most associated with the type of positive or negative stimuli administered to the donkeys during the two test sessions, as shown in Figure 4. The sum of the first three PCs reached 83.30% of the variability, which is indicative of a good descriptive capability of the analysis. The scores projected along the PC1 differed significantly between the two study sessions (V = 45, *p* = 0.004), while those associated with PC2 and PC3 were non-significant (V = 22, *p* = 1, and V = 9, *p* = 0.130, respectively). The grouping by test type was mainly represented on the PC1 (which accounts for 58.00% of the total observed variability), with the subjects’ scores mainly distributed on the left side in the case of the (+) test, and on the right side in the case of the (−) test. The Spearman correlation coefficients calculated between the PC1 and the behaviours included in the analysis allowed for the identification of two groups of opposed modes. During the (+) test, in the presence of rewards, animals mostly manifested both ears side-up (*r*_L-ear SU_ = −0.793, *p* < 0.0001; *r*_R-ear SU_ = −0.585, *p* = 0.011), the right ear side-down (*r*_R-ear SD_ = −0.497, *p* = 0.035), and the standing position maintained on the four legs (*r*_4 legs_ = −0.798, *p* < 0.0001). Conversely, during the (−) test, following the administration of the frustrating stimulus, donkeys kept both ears front-up (*r*_L-ear FU_ = 0.924, *p* < 0.0001; *r*_R-ear FU_ = 0.946, *p* < 0.0001). Moreover, they bent the knee by lifting a front leg repeatedly (*r*_bent knee_ = 0.656, *p* = 0.003), showed an increased tendency to walk (*r*_walk_ = 0.709, *p* = 0.001), and walk away (*r*_walk away_ = 0.578, *p* = 0.012) from the manipulandum.

**Figure 5:**
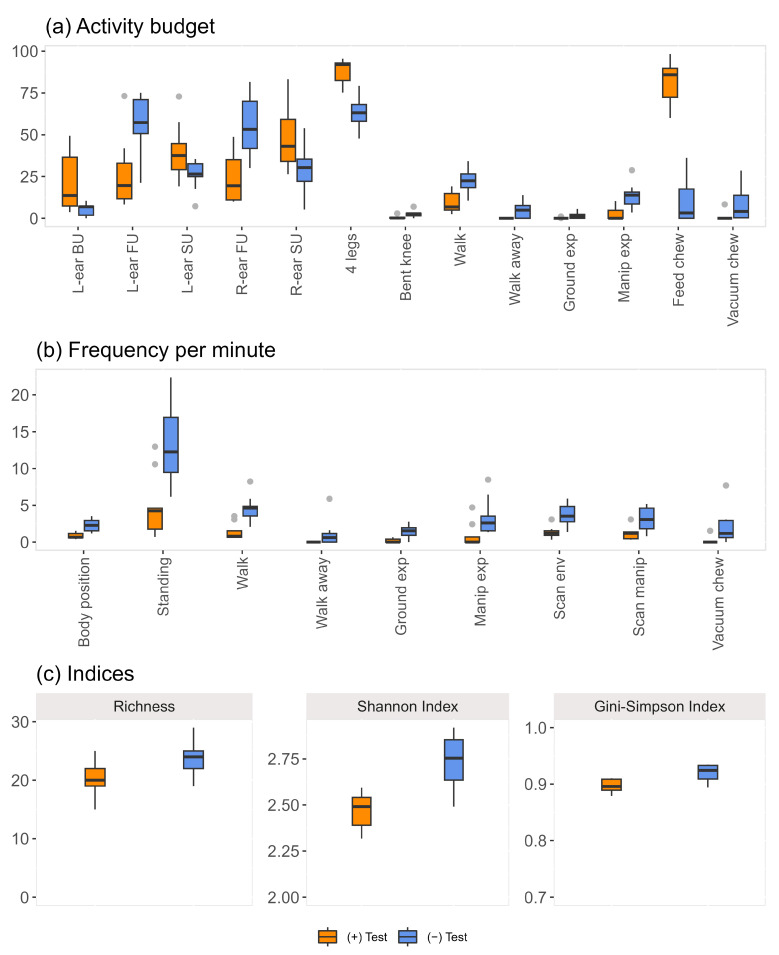
Graphical representation of each behaviour/behavioural modes distributions calculated for the following behavioural diversity indices: (**a**) activity budget (percentage of time spent by subjects in manifesting each behaviour); (**b**) frequency per minute (number of events per minute): the possible variants of some behaviours (i.e., body position, standing, ground exploration) have been grouped and considered together, following what is reported in Table 3; (**c**) richness (count of the different behavioural modes); Shannon index (behavioural richness and evenness); Gini-Simpson index (probability that two behaviours randomly selected from a sample will be different). Only significant comparisons are shown (*p* < 0.05), for the complete list of behaviours see Supplementary Table S1.

The percentage of time the subjects were engaged in different behaviours significantly differed between the two test sessions and reflects, to a good extent, the results obtained through the PCA (Figure 5a).

Notably, time spent with the left ear in the backup position (V_L-ear BU_ = 45, *p* = 0.004) and both ears side up (V_L-ear SU_ = 45, *p* = 0.004; V_R-ear SU_ = 41, *p* = 0.027) was significantly higher during the (+) test (in presence of the rewarding stimulus). We found the same significant result for time spent standing on all four legs (V_4 legs_ = 45, *p* = 0.004) and feed chewing (V_Feed chew_ = 45, *p* = 0.004). Instead, both ears in the front-up position (V_L-ear FU_ = 0, *p* = 0.004; V_R-ear FU_ = 0, *p* = 0.004) and avoidance behaviours like walking and walking away from the manipulandum (V_Walk_ = 0, *p* = 0.004; V_Walk away_ = 0, *p* = 0.036) were performed more often during the (−) test. Other behaviours likely associated with frustration and performed significantly often in the (−) test were exploration aptitude towards both the ground and the button (V_Ground exp_ = 0, *p* = 0.036; V_Manip exp_ = 0, *p* = 0.004), a repeatedly bent knee (V_Bent knee_ = 0, *p* = 0.014), and vacuum chewing (V_Vacuum chew_ = 0, *p* = 0.022).

A higher number of changes per minute in the animal position was observed during the (−) test (Figure 5b). These include more frequent changes in body position (front, left and right position with respect to the manipulandum; V_Body position_ = 0, *p* = 0.022) and limb position (3 legs, 4 legs, bent knee and pointing; V_Standing_ = 1, *p* = 0.008). Moreover, our results also highlighted a higher frequency of behavioural changes (Figure 5b). These include walking and walking away (V_Walk_ = 0, *p* = 0.004; V_Walk away_ = 0, *p* = 0.036), exploration of both the environment and the manipulandum (V_Ground exp_ = 0, *p* = 0.022; V_Manip exp_ = 1, *p* = 0.013; V_Scan env_ = 1.5, *p* = 0.025; V_Scan manip_ = 0, *p* = 0.022), and vacuum chewing (V_Vacuum chew_ = 0, *p* = 0.022).

The three additional indices (i.e., richness, Shannon’s, and Gini-Simpson indices) that were calculated to get a general overview of the levels of behavioural diversity also showed significantly higher values in the context of the (−) test (Figure 5c). The richness, which is the count of the different behavioural types recorded, is indicative of a richer behavioural repertoire in the presence of an undesired stimulus (V_Richness_ = 0, *p* = 0.022). The higher Shannon’s index during the (−) test reflects a larger number of behaviour types and more equal abundances in the time spent by the donkeys in performing them (V_Shannon Index_ = 3, *p* = 0.020), that is, there were no dominant behaviours over others, but all were similarly expressed in terms of percentage time. Since the latter is not quantitatively easy to interpret, the Gini-Simpson was considered, and it can be interpreted as the probability that two behaviours randomly selected from a sample would be different. Although the diversity levels were elevated in both sessions (median_(+)test_ = 0.896, interquartile range = [0.889; 0.909]; median_(−)test_ = 0.924, interquartile range = [0.909; 0.933]), which is indicative of a general wide variety of behaviours in terms of both richness and evenness, significantly higher values were associated with the (−) test (V_Gini-Simpson Index_ = 4, *p* = 0.027), again reflecting a greater behavioural complexity in the context of a negative stimulus.

### 3.2. Heart Rate and Heart Rate Variability

In general, HR correlated negatively with the HRV parameters (r_HR_ vs. _RR_ = −1, *p* < 0.0001; r_HR_ vs. _rMSSD_ = −0.321, *p* = 0.032; r_HR_ vs. _Std RR_ = −0.370, *p* = 0.013), as expected [52]. Medians and interquartile ranges of RR, Std RR, HR, and rMSSD, calculated for each phase of the two test types, are reported in Table 5. When considering the two study sessions ((+) and (−)) separately, no significant differences were found among the ‘pre’, ‘test’, and ‘post’ phases in the mean RR, HR, and Std RR distributions. Conversely, the average rMSSD result was significantly lower during the ‘post’ phase of the negative session compared to both the ‘pre’ and ‘test’ phases of the same session (t_pre(−)_ vs. _post(−)_ = 4.901, *p* = 0.002; t_test(−)_ vs. _post(−)_ = 3.532, *p* = 0.011), and also with respect to the ‘post’ phase of the positive session (t_post(+)_ vs. _post(−)_ = 5.085, *p* = 0.002).

## 4. Discussion

### 4.1. Facial Expressions and Postures

From the analysis of the behavioural observations recorded during the (+) and (−) tests, which were designed to induce in animals respectively a state of ‘euphoria’ and ‘frustration’, it emerged that there are facial expressions, postures, and behaviours that can potentially be indicative of the two opposing situations. In particular, one of the most important variables identified by PCA was the position of the ears.

The posture of the ear has been proposed as an indicator of the emotional state in large animals, especially in those that have a limited range of facial movements to produce distinguishable facial expressions [29]. In Equidae, it has been found that the way horses place their ears is a good indicator of their perception of a situation as ‘positive’ or ‘negative’ [61,62]. By our results, the ears were held for a longer time in the upright frontal position during the presumed negative emotional state. This has also emerged in two previous studies conducted on sheep [44] and donkeys [30]. In such studies, ears kept in a frontal position were related to a state of alertness in these species. This emotional state of alertness, in our setting, can be due to the fact that the animals did not expect the stones as a reward following the key pressing (reaction to a novel object/unexpected situation), and also likely to the noise caused by their falling into the manipulandum tray. As will be discussed later in reference to the frustration-exploration hypothesis [63], this state of alertness is not necessarily negative per se, as it represents the animal’s response to the unexpected and potentially frustrating situation it is faced with (i.e., failure to obtain the reward). Raising the alertness threshold may represent the first step of a series of exploratory behaviours and new attempts aimed at obtaining the expected reward. However, recognizing the state of alertness in donkeys may indicate that something is perceived negatively or not indulging their needs.

On the other hand, the ears held sideways were more manifested in emotionally positive contexts. This result confirms what is also observed in other species, such as sheep [44,64] and horses [65]. As for the backwards-up ear orientation, in line with what was previously observed in sheep [44] and horses [20], our results highlighted a significant difference between the two emotional contexts. In fact, only the orientation of the left ear was significantly associated with the (+) test; however, the backwards-up position of the right ear was also concurrently observed during the (+) test, although the number of animals included in the study was likely not enough to obtain a robust estimate of the statistical significance for this model. Conversely, the ears kept ‘back down’, against the head did not show any difference in their manifestation in the two emotional contexts. Concerning the frequency of ear orientation changes, we did not find any significant evidence of increasing values in negative situations, as already found in sheep [44,66]. In donkeys, according to an earlier study, ear movements are frequent and more related to a negative emotional state [29]. Here, we observed a slightly increased frequency in the (−) test, even though the difference from the positive one was not substantial.

As for body postures, donkeys tended to distribute their body weight on all four legs longer during a pleasant situation (up to an average of more than 90% of the time). On the other hand, in a frustrating emotional situation, animals kept one of their forelimbs bent for longer. The increased time budget spent with a bent knee was made up of leg lifting movements related to increased restlessness and agitation. In addition, the frequency per minute of the change of the standing posture and the body orientation towards the manipulandum was significantly higher in the (−) vs. (+) test. This, together with the enhanced budget of time and the higher frequency per minute on behaviours related to movement (walking and walking away) and exploration (i.e., ground and manipulandum exploration), indicate a greater ‘restlessness’ of the animals during the negative emotional situation, compared to the positive one. In addition, donkeys appear to look around more often in the negative test, as well as looking more at the manipulandum. In their Delphi consultation, Pannewitz and Loftus [67] have recently attempted to define behavioural indicators of frustration in horses: improved movement and locomotion have been found to be recurrent, in accordance with our results. However, in their conclusions, they underline the need to define behaviours that can be a reliable indicator of equine frustration. Here, we tried to cast light on this aspect and fill this gap by highlighting behaviours we found to be related to this negative emotion in the donkey species (an equid on which the literature is even more scarce compared to the horse). Obviously, the fact that the animals were engaged in eating the reward during the (+) test influenced the difference in behavioural responses between the (+) and (−) tests. However, as just discussed, the behaviours manifested during the (−) test could be indicative of frustration.

Feed chewing was more documented in the (+) test, as might be expected, given the nature of the reinforcement. This happened although the usual feed (hay) was present on the ground even during the negative test. During the negative test, hay attracted the interest of only a few individuals, while the others were repeatedly pressing the manipulandum’s button or spent more time looking around or leading exploratory behaviours. The result of enhanced exploratory behaviours during the ‘frustrative’ condition supports the frustration-exploration hypothesis; this theory posits that a frustrative non-rewarding condition (i.e., the occurrence of non-reward in a context that has been previously associated with a reward) leads to exploration [63]. The primary function of enhanced investigation is to broaden the extent of response selection. Thus, in this perspective, frustration not only compels the individual to explore alternative responses, but also draws its awareness towards ignored features of the environment [63].

During the (−) test, the frequency per minute and the budget of time spent manifesting the vacuum chewing increased. This behaviour in the literature is generally associated with a state of stress in animals and it is especially manifested in contexts of frustration in horses [50,68,69,70]. Indeed, the vacuum chewing, as well as the snort, can be considered displacement activities. These are defined as activities that occur in circumstances where they are seemingly irrelevant to current events and appear to reflect the motivational ambivalence/frustration deriving from conflict situations [71,72,73].

Moreover, in the analysis of behaviour, we considered lateralization as a possible indicator of animal emotion. In fact, cerebral lateralization has been recently studied in several animal species, including donkeys [74], and has been related to the valence of emotion that the animal is experiencing, according to the emotional-valence hypothesis [75]. This hypothesis suggests that negative emotions are predominantly processed by the right hemisphere, while positive ones are processed by the left hemisphere. Therefore, a manifested lateralization pattern (e.g., of the body or the head in front of a stimulus) could give insights into an animal’s emotional processing. In our study, no evidence of lateralized body or head postures has been shown by our experimental design. Indeed, no significant difference was found in the body’s and/or head’s orientation toward the manipulandum (which was the one providing the reinforcement/stimulus) between the positive and negative situations. However, analysis of movements of the limbs comparing a positive and a negative condition could give some insights about this topic in future studies to confirm the signs of laterality in the form of limb preference in donkeys, as highlighted by Zucca et al. [74].

Nowadays, enhanced behavioural diversity is typically considered a positive welfare indicator in various species [31,76,77]. It is reasonable to expect increased behavioural diversity, whether seen from the perspective of animals living in an environment rich in stimuli rather than a deprived one. However, its interpretation may change if it is seen from the point of view of positive/negative emotions. Indeed, in a stimulating environment where individuals do not show signs of apathy, a negative emotional context (i.e., not optimal from the point of view of animal welfare), such as the one of frustration, may increase the complexity and behavioural diversity. On the other hand, a positive emotional context, which is likely more stable and predictable by the individual, may lead to a lesser variety of manifested behaviours. In the present study, a greater significant behavioural complexity was shown in the condition with the frustrating stimulus. This supports the hypothesis of individuals trying to cope with frustrating and unexpected situations by manifesting more inquiring behaviours and being more restless. Thus, in this case, behavioural diversity can be considered as a stress indicator, rather than a measure of the levels of animal welfare, which highlights the opportunity to further investigate alternative applications and interpretations of these indices, as already highlighted by Cronin and Ross [78] in their note. In fact, as discussed by these authors, the common practice of excluding some behavioural categories or not considering the valence of behaviours can easily deviate the results interpretation of behavioural diversity indices. Furthermore, as seen in this case, the assumption that larger behavioural repertoires reflect better welfare is sometimes questionable [78].

### 4.2. HRV Analysis

As for HRV analysis, the only parameter that varied significantly when comparing the (+) and (−) tests and their respective phases (pre, test, and post) was rMSSD, a parameter reflecting short-term changes in HRV due mainly to parasympathetic nervous system activity [34,51]. rMSSD was significantly lower in the ‘post’ (−) test phase than in both the ‘pre’ (−) test/(−) ‘test’ phases, and the ‘post’ (+) test phase. Essentially, this difference could reflect a less pronounced shift toward vagal tone occurring after the negative test. This is consistent with restlessness behaviours shown during the (−) test and with the frustration-exploration hypothesis which could be related to increased arousal in the animal. In fact, HRV parameters are indicative of emotional arousal rather than emotional valence [79]. Nevertheless, it has been hypothesized that rMSSD values show higher values during positive emotional states, and lower values (corresponding to parasympathetic deactivation) during negative states, both in humans and animals [7,80]. Regarding frustration specifically, rMSSD values have been shown to be associated with long-term frustration levels in humans [81], but not, for example, in dwarf goats facing a frustrating situation [82]. Therefore, it would be interesting to further investigate the association between rMSSD and frustration in equine species, possibly through the concurrent exploration of multiple indicators, which should be useful in achieving an interpretation of the internal state of the animal [5].

### 4.3. Limitations of the Study

This study has some limitations to consider, starting from the selected sample, since its small size precludes a generalization of our conclusions to the whole species. Moreover, the different proportion between sexes (six females and three males) and the fact that the donkeys are non-working and non-productive animals, but rather animals that are involved in AAI, can represent another limit to the possibility of generalizing the results. A second limitation is related to the choice of the feed reward as the type of stimulus provided, as it limits the investigation of facial expressions to the upper facial part, excluding the mouth/nostrils area. Nevertheless, the feed reward allowed us to assume, with some confidence, the kind of emotion induced in donkeys (and thus to distinguish between (+) and (−) situations), and to make observations on ear movements and positions, which have already been proposed as emotional indicators in equids. Moreover, the presence or absence of food respectively during the (+) and (−) tests could also affect the manifestation of vacuum chewing. However, this behaviour was also rarely observed during the positive test, and it could be distinguished from normal food chewing as it occurred at times when food was not yet available inside the tray.

Another limitation is related to the experimental setting, as a number of environmental factors may have influenced the behavioural and physiological responses of the donkeys. As far as possible, however, an attempt to minimise the influence of some confounding variables was made (e.g., choice of the time of day to carry out the experiment, control over the presence of other people and possible sources of external noise, etc.).

Concerning the analysis of HRV, the Polar V800 has been recently validated for its use in horses [49], but not yet in donkeys. In our case, it was not possible to carry out this validation; moreover, some data were discarded due to the poor quality of the signal collected. In addition, given the experimental design that involved tests of short duration and because it is inadvisable to compare segments of different duration [83], 5 min segments were not used as usually recommended [83]. On the contrary, based on what was reported by other authors, ultra-short segments of one minute were considered [33,84]. Other unresolved issues concerning the analysis of HRV in donkeys, which is still in its infancy, were previously discussed in a scoping review on HR and HRV in donkeys [34]. This review also revealed that only two other studies have so far analysed HRV in donkeys [35,36], which makes our results difficult to compare and, thus, need to be interpreted with caution. However, our investigation can be considered a pilot study and it is one of the first to report data on HRV in this species.

## 5. Conclusions

Recognizing and understanding donkeys’ emotions is critical to their welfare and can benefit the animal itself, the human-animal relationship, and the environment in which the animals live (e.g., housing and husbandry practices). In this study, donkeys confronted with two situations of presumed different emotional content (positive vs. negative) showed different behavioural and physiological responses. In particular, ear position and orientation, body position and postural changes, and exploratory behaviours were significantly different between the two situations.

Overall, the authors interpreted the behaviour manifested during the negative situation (i.e., ears mainly erected frontally, repeatedly bent knee, and frequent changes in body position) as attributable to a situation of frustration and exploration, in which the animal’s attention is triggered by not receiving the expected reward. This interpretation would also be compatible with the HRV results, in which the lower rMSSD resulting after the negative test could be related to an increased state of arousal in the animals. Nevertheless, the HRV analysis in this species still needs to be consolidated through the use of a more robust methodology and validated devices.

Although the small sample size considered in this study precludes drawing firm conclusions, these results provide information about an equine species that, despite its wide distribution, is so far understudied, and cast light on possible indicators that can be considered in further research to better understand the emotional state of donkeys.

## Figures and Tables

**Table 1:** Donkeys’ inclusion/exclusion criteria.

Inclusion Criteria	Exclusion Criteria
Clinically healthy subjects	Individuals with physical or behavioural pathologies
Subject with typical behavioural development	Lactating individuals, females in the last third of pregnancy or in oestrous
Subjects used to contact with humans	Subjects not socialised with humans
Females or geldings	Stallions
Young and adult individuals (age > 12 months)	Subjects with a history of abuse

**Table 2:** Training and testing daily program.

Day	Activity
0	Acclimatization
1–7	Training sessions
8	Positive test
9	Negative test

**Table 3:** Operational definition, type of event recorded, and acronyms of the considered behaviours.

Behaviour	Variants	Acronym	Description	Event Type
Push			The complete pressure of the buzzer. This allows the animal to obtain the reinforcement, with the button starting to blink and making a sound indicating the proper pressure.	Point event
Attempt			The partial pressure of the button. The animal demonstrates intentionality in pressing the button, however resulting to be too light (with no trigger of the buzzer sound and light) or in the wrong place (e.g., on the coloured border surrounding the button).	Point event
Bite			The animal bites a part of the manipulandum.	Point event
Kick			The animal kicks against the manipulandum or towards other donkeys on the sides of the test area.	Point event
Walk		Walk	Movement from one point to another, with the execution of at least one complete step (movement of all 4 legs).	State event
Standing	3-legs	3-legs	The animal stands still (or performs less than 1 complete step). 4-legs: standing with weight-bearing on all 4 limbs with no preferred loading; 3-legs: the weight-bearing is on 3 limbs with a hind limb resting; pointing: the animal places a foot forwards outside of the main body frame (minimum 1 hoof length) with reduced weight-bearing; bent-knee: the animal has one of the front legs with the knee bent and that not bears weight. The standing position variants are graphically presented in Figure 3.	State event
	4-legs	4-legs		
	bent knee	Bent knee		
	pointing	Pointing		
Walk away		Walk away	The animal loses interest in the manipulandum and walks away from it.	State event
Pawing			Pawing with the forelimbs/investigating with the paw.	Point event
Stomp			One foreleg is raised and lowered sharply and firmly against the ground. Stomping differs from pawing in that it is a vertical rather than horizontal movement of the leg.	Point event
Ground exploration	ground	Ground exp	The donkey sniffs the ground (when the donkey moves to sniff somewhere else, the walk is still counted). Ground: the donkey does not eat while sniffing on the ground; ground eating: the animal feeds off the ground while exploring.	State event
	ground eating	Ground eat		
Area exploration			The animal sniffs/explore other parts of the test area (i.e., fence, straps, iron grid).	State event
Manipulandum exploration		Manip exp	The animal sniffs/explore parts of the manipulandum.	State event
Stones exploration			The animal sniffs/explore the stones (i.e., the negative reinforcement).	State event
Scanning environment		Scan env	The donkey looks towards its surroundings. Only the position/direction of the head with respect to the manipulandum is considered.	State event
Scanning manipulandum		Scan Manip	The donkey looks towards the manipulandum (covered or uncovered). Only the position/direction of the head with respect to the manipulandum is considered.	State event
Snort			Forceful exhalation through the nostrils and characterized by an audible flutter pulsation.	Point event
Vocalizations			Other type of sounds produced by the donkey and different from a snort.	Point event
Tray removal			The donkey removes/detaches the tray from the manipulandum.	Point event
Body position	frontal	Body F	The donkey body position with respect to the manipulandum.	State event
	left	Body L		
	right	Body R		
Head position	frontal	Head F	The donkey head position with respect to the manipulandum.	State event
	left	Head L		
	right	Head R		
Left ear position	back-up	L-ear BU	Position of the left ear as described in Figure 2.	State event
	back-down	L-ear BD		
	side-up	L-ear SU		
	side-down	L-ear SD		
	front-up	L-ear FU		
Right ear position	back-up	R-ear BU	Position of the right ear as described in Figure 2.	State event
	back-down	R-ear BD		
	side-up	R-ear SU		
	side-down	R-ear SD		
	front-up	R-ear FU		
Feed chewing		Feed chew	The donkey eats and chew feed.	State event
Vacuum chewing		Vacuum chew	Chewing with nothing in the mouth.	State event
Mouth movements		Mouth mov	Unusual movements of the mouth that are not related to feed chewing.	State event
Yawn			The donkey yawns.	Point event
Flehmen			The upper lip curls back to expose gums with incisors meeting, head tips back and rapidly points muzzle upwards.	Point event

**Table 4:** List of behavioural diversity indices, their description, and the possible range of values.

Index	Description	Numerical Range [Min; Max]
Behavioural frequencies	Number of behavioural variants manifested per minute for each type of behaviour.	[0; +∞]
Activity budgets	The percentage of time spent by donkeys in manifesting each behavioural variant.	[0; 100]
Richness	The count of the different behavioural types exhibited by the animals in the corresponding ethogram.	[0; +∞]
Shannon’s diversity index	Nonlinear index that considers both the behavioural richness and evenness. Higher index values reflect a larger numbers of behaviour types and more equal abundances.	[0; +∞]
Gini-Simpson index	A diversity index that measures the probability that two behaviours randomly selected from a sample will be different.	[0; 1]

**Table 5:** Median and interquartile ranges [1st quartile; 3rd quartile] of HRV parameters recorded in donkeys before (pre), during (test), and after (post) the positive (+) and negative (−) test sessions.

	(+) Test			(−) Test		
	Pre ^a^	Test ^a^	Post ^b^	Pre ^a^	Test ^b^	Post ^a^
RR (ms)	1116.60 [998.0; 1187.80]	1026.00 [944.40; 1119.10]	1086.00 [896.00; 1182.00]	1218.00 [1042.10; 1288.70]	1122.80 [979.10; 1147.00]	1079.00 [1007.20; 1150.70]
Std RR (ms)	102.68 [88.72; 127.27]	139.90 [133.74; 169.68]	122.39 [84.77; 169.87]	104.39 [90.62; 129.89]	128.02 [109.98; 192.84]	118.29 [51.72; 141.70]
HR (beats/min)	53.74 [50.59; 60.25]	58.49 [53.65; 63.69]	55.26 [50.76; 67.20]	49.26 [46.59; 57.58]	53.44 [52.31; 61.68]	55.62 [52.14; 59.57]
rMSSD (ms)	94.00 [90.42; 112.88]	127.77 [88.45; 150.37]	120.38 [114.98; 155.50]	99.90 [92.20; 133.70]	125.43 [103.68; 172.36]	62.85 [49.18; 86.67]

^a^ Missing data for one subject; ^b^ Missing data for two subjects.

## Data Availability

The data presented in this study are available on request from the corresponding author.

## Supplementary Materials

The following supporting information can be downloaded at: https://www.mdpi.com/article/10.3390/ani13091466/s1, Figure S1: Graphical representation of state and point behaviours recorded for each donkey during the (+) and (−) tests; Table S1: Behavioural modes and positions observed during the (+) and (−) tests. Median [1st quartile; 3rd quartile] of the activity budget, frequency per minute, richness, Shannon and Gini-Simpson indices. Wilcoxon signed-rank test statistic (V) and *p*-values in bold: significant differences between the two test groups.
