# Supplementary material for: A Pilot Study on Behavioural and Physiological Indicators of Emotions in Donkeys
Source: Animals (Basel). 2023 Apr 25;13(9):1466. doi: 10.3390/ani13091466 (PMC10177292; doi:10.3390/ani13091466)
Supplement: Supplementary file 1 [file animals-13-01466-s001.zip › animals-2313160-Table S1-proofread.pdf]

animals

Table S1. Behavioural modes and positions observed during the (+) and (−) tests. Median [1st quartile; 3rd quartile] of the activity budget, frequency per minute, richness, Shannon and Gini-Simpson indices. Wilcoxon signed-rank test statistic (V) and *p*-values in bold: significant differences between the two test groups.

Type	Mode	(+) Test	(−) Test	Wilcoxon signed-rank test
Activity budget	Body F	43.652 [0.000; 83.133]	8.057 [1.806; 25.892]	V = 32, p = 0.301
	Body L	0.000 [0.000; 64.507]	27.925 [0.000; 41.135]	V = 15, p = 0.933
	Body R	0.000 [0.000; 0.000]	29.581 [0.000; 53.305]	V = 3, p = 0.142
	Head F	35.850 [31.423; 37.743]	26.280 [22.556; 36.482]	V = 34, p = 0.203
	Head L	41.856 [39.524; 51.949]	25.150 [12.384; 47.195]	V = 33, p = 0.250
	Head R	6.848 [6.072; 19.437]	28.398 [21.125; 31.084]	V = 9, p = 0.129
	L-ear BU	13.652 [7.347; 36.564]	6.908 [1.855; 7.199]	**V = 45, p = 0.004**
	L-ear BD	2.454 [0.567; 3.741]	0.883 [0.000; 7.389]	V = 22, p = 1
	L-ear FU	19.638 [11.736; 32.948]	57.288 [50.748; 71.007]	**V = 0, p = 0.004**
	L-ear SD	0.497 [0.000; 2.287]	3.446 [0.135; 5.832]	V = 14, p = 0.624
	L-ear SU	37.556 [29.141; 44.717]	26.536 [24.919; 32.593]	**V = 45, p = 0.004**
	R-ear BU	16.820 [3.409; 29.037]	5.058 [3.782; 8.854]	V = 39, p = 0.055
	R-ear BD	2.891 [0.585; 4.309]	1.764 [0.000; 3.037]	V = 24, p = 0.441
	R-ear FU	19.515 [11.035; 35.133]	53.243 [41.826; 69.999]	**V = 0, p = 0.004**
	R-ear SD	0.405 [0.000; 22.030]	0.000 [0.000; 1.150]	V = 22, p = 0.205
	R-ear SU	43.138 [34.102; 59.218]	30.331 [22.130; 35.446]	**V = 41, p = 0.027**
	3 legs	0.000 [0.000; 0.879]	1.438 [0.603; 2.236]	V = 6, p = 0.205
	4 legs	92.017 [82.508; 92.907]	63.110 [58.107; 68.095]	**V = 45, p = 0.004**
	Bent knee	0.164 [0.000; 0.522]	2.765 [1.510; 3.000]	**V = 0, p = 0.014**
	Pointing	0.000 [0.000; 0.000]	0.000 [0.000; 0.000]	V = 4, p = 0.789
	Walk	6.784 [4.959; 14.802]	22.441 [18.423; 26.595]	**V = 0, p = 0.004**
	Walk away	0.000 [0.000; 0.000]	4.912 [0.000; 7.618]	**V = 0, p = 0.036**
	Ground exp	0.000 [0.000; 0.000]	1.119 [0.000; 2.368]	**V = 0, p = 0.036**
	Ground eat	0.000 [0.000; 0.000]	0.000 [0.000; 2.610]	V = 1, p = 0.201
	Manip exp	0.000 [0.000; 4.793]	13.876 [8.533; 15.657]	**V = 0, p = 0.004**
	Scan env	3.546 [3.172; 3.924]	12.531 [7.515; 26.730]	V = 9, p = 0.129
	Scan manip	3.184 [1.891; 7.843]	9.420 [5.318; 12.674]	V = 14, p = 0.359
	Mouth mov	0.000 [0.000; 0.000]	0.000 [0.000; 2.274]	V = 4, p = 0.418
	Feed chew	85.865 [72.390; 89.766]	3.246 [0.000; 17.522]	**V = 45, p = 0.004**
	Vacuum chew	0.000 [0.000; 0.000]	4.137 [0.300; 13.775]	**V = 0, p = 0.022**
Frequency per minute	Body position	0.649 [0.588; 1.176]	2.273 [1.538; 2.941]	**V = 0, p = 0.022**
	Head position	15.862 [12.727; 21.176]	12.308 [11.176; 14.545]	V = 27, p = 0.652
	L-ear position	18.636 [16.471; 20.000]	23.636 [17.059; 28.235]	V = 14, p = 0.359
	R-ear position	18.636 [11.176; 24.706]	20.000 [19.412; 26.364]	V = 15, p = 0.426
	Walk	0.789 [0.649; 1.538]	4.615 [3.553; 4.865]	**V = 0, p = 0.004**
	Walk away	0.000 [0.000; 0.000]	0.606 [0.000; 1.176]	**V = 0, p = 0.036**
	Standing	4.242 [1.765; 4.615]	12.273 [4.615; 16.923]	**V = 1, p = 0.008**
	Ground exp	0.000 [0.000; 0.395]	1.538 [0.909; 1.946]	**V = 0, p = 0.022**
	Manip exp	0.000 [0.000; 0.690]	2.595 [1.538; 3.529]	**V = 1, p = 0.013**
	Scan env	1.212 [0.909; 1.538]	3.529 [2.763; 4.848]	**V = 1.5, p = 0.025**
	Scan manip	1.176 [0.455; 1.379]	3.077 [1.818; 4.615]	**V = 0, p = 0.022**
	Mouth mov	0.000 [0.000; 0.000]	0.000 [0.000; 0.909]	V = 1, p = 0.106
	Feed chew	0.690 [0.588; 1.176]	0.324 [0.000; 1.538]	V = 16, p = 0.834
	Vacuum chew	0.000 [0.000; 0.000]	1.176 [0.606; 2.941]	**V = 0, p = 0.022**
Indices	Richness	20.000 [19.000; 22.000]	24.000 [22.000; 25.000]	**V = 0, p = 0.022**

Type	Mode	(+) Test	(−) Test	Wilcoxon signed-rank test
	Shannon Index	2.491 [2.390; 2.541]	2.754 [2.635; 2.854]	**V = 3, *p* = 0.020**
	Gini-Simpson Index	0.896 [0.889; 0.909]	0.924 [0.909; 0.933]	**V = 4, *p* = 0.027**
