# Supplementary figures and images for: A Pilot Study on Behavioural and Physiological Indicators of Emotions in Donkeys
Source: Animals (Basel). 2023 Apr 25;13(9):1466. doi: 10.3390/ani13091466 (PMC10177292; doi:10.3390/ani13091466)

Euclidean

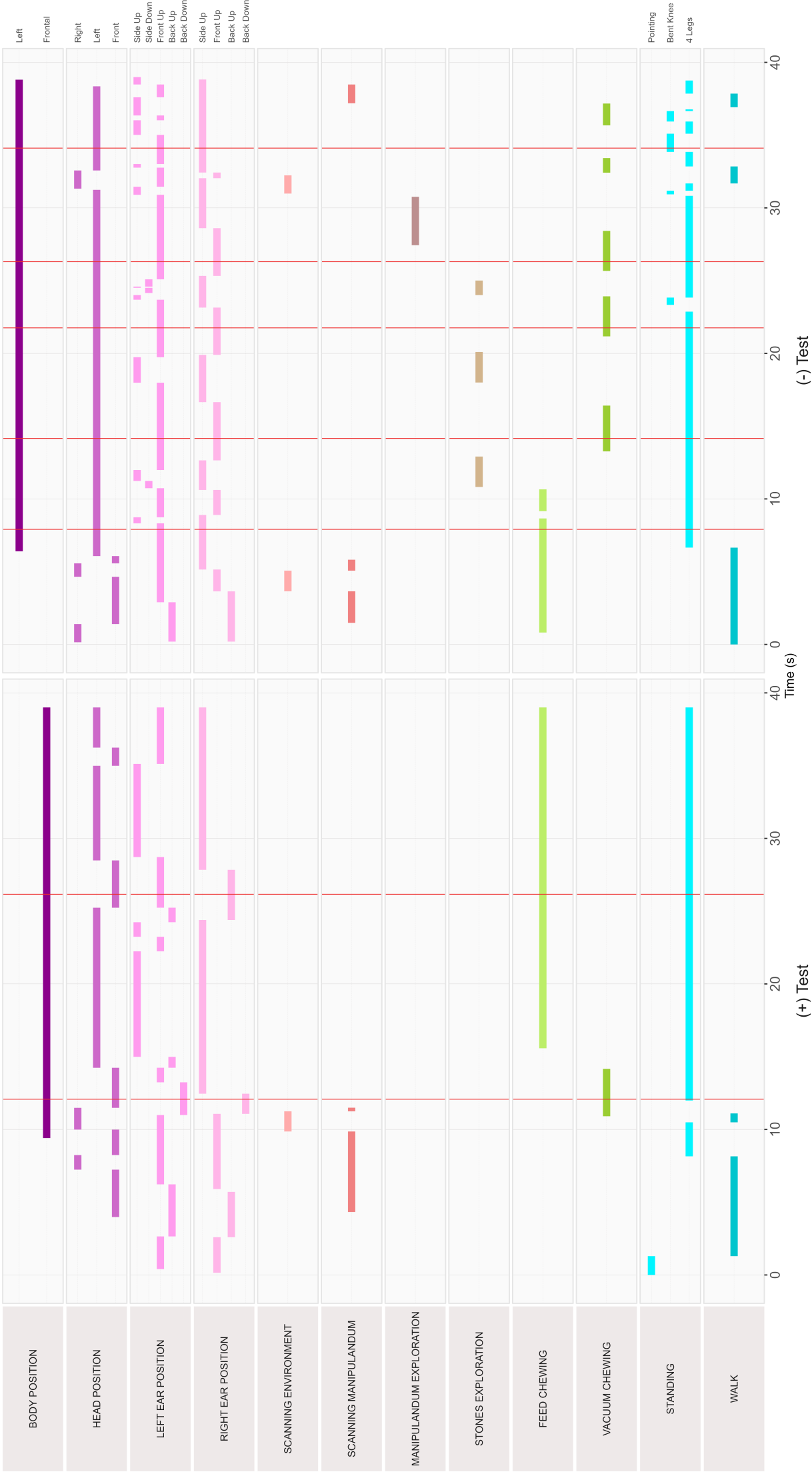

Giorgina

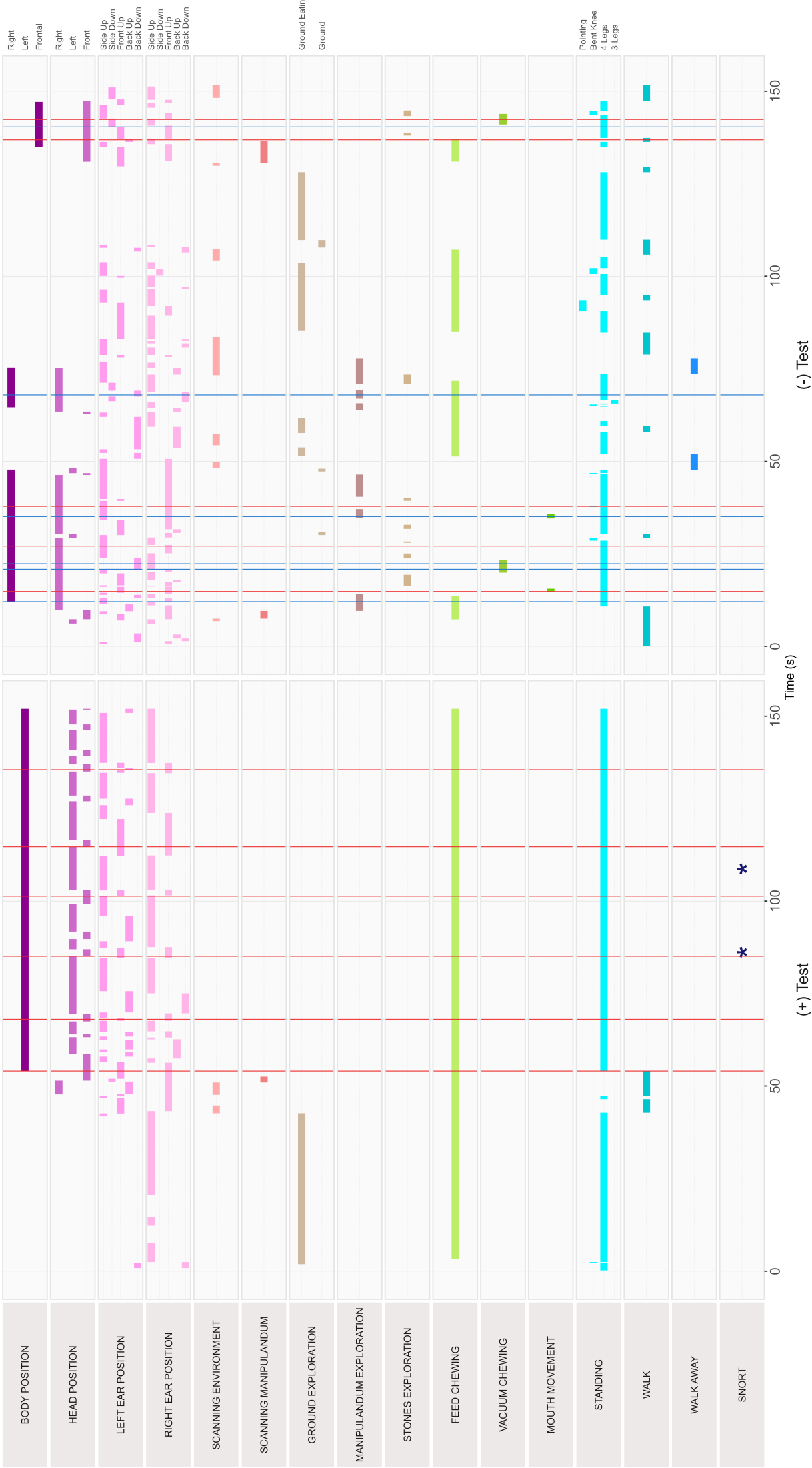

Huggy

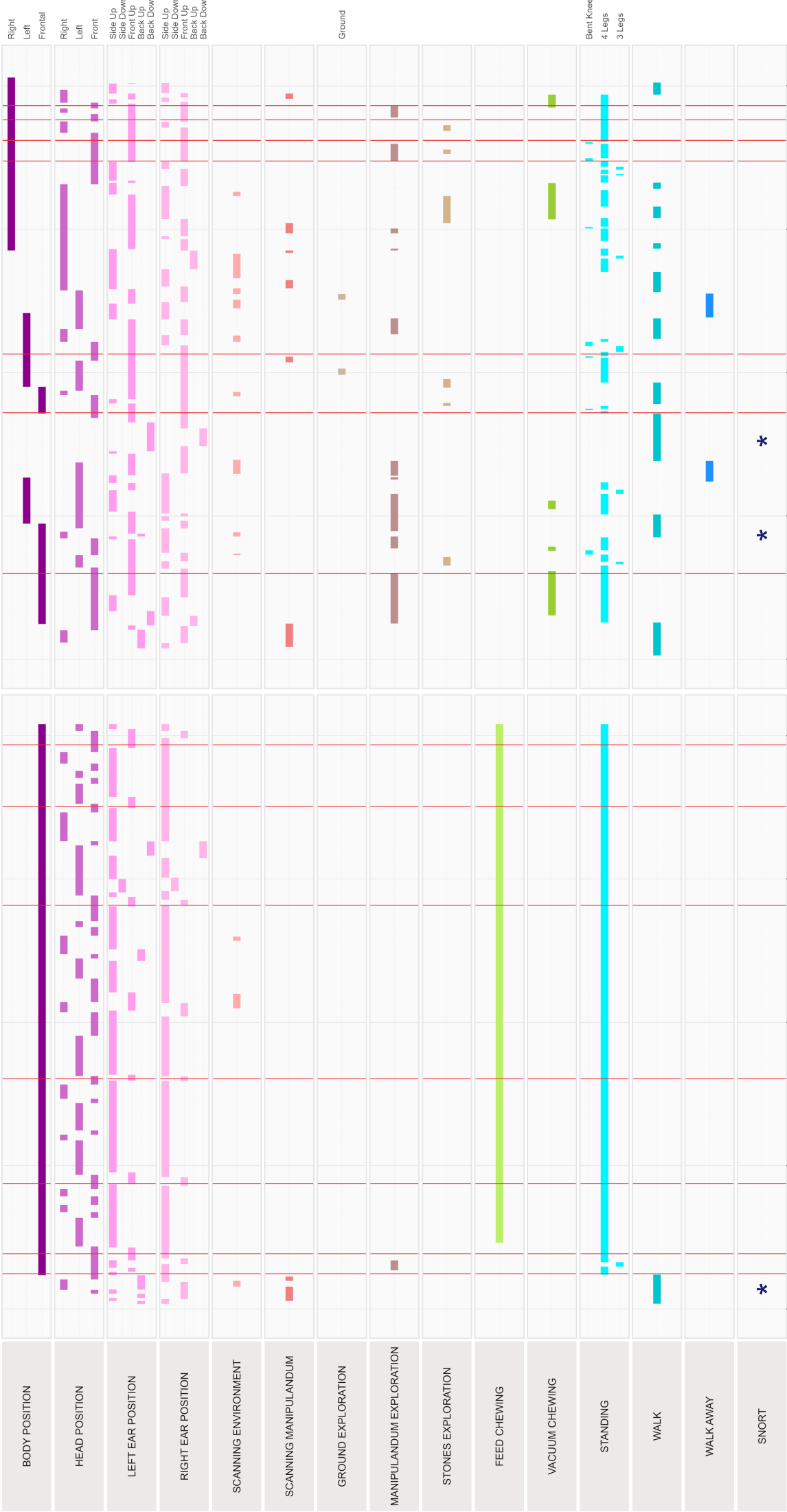

Lolita

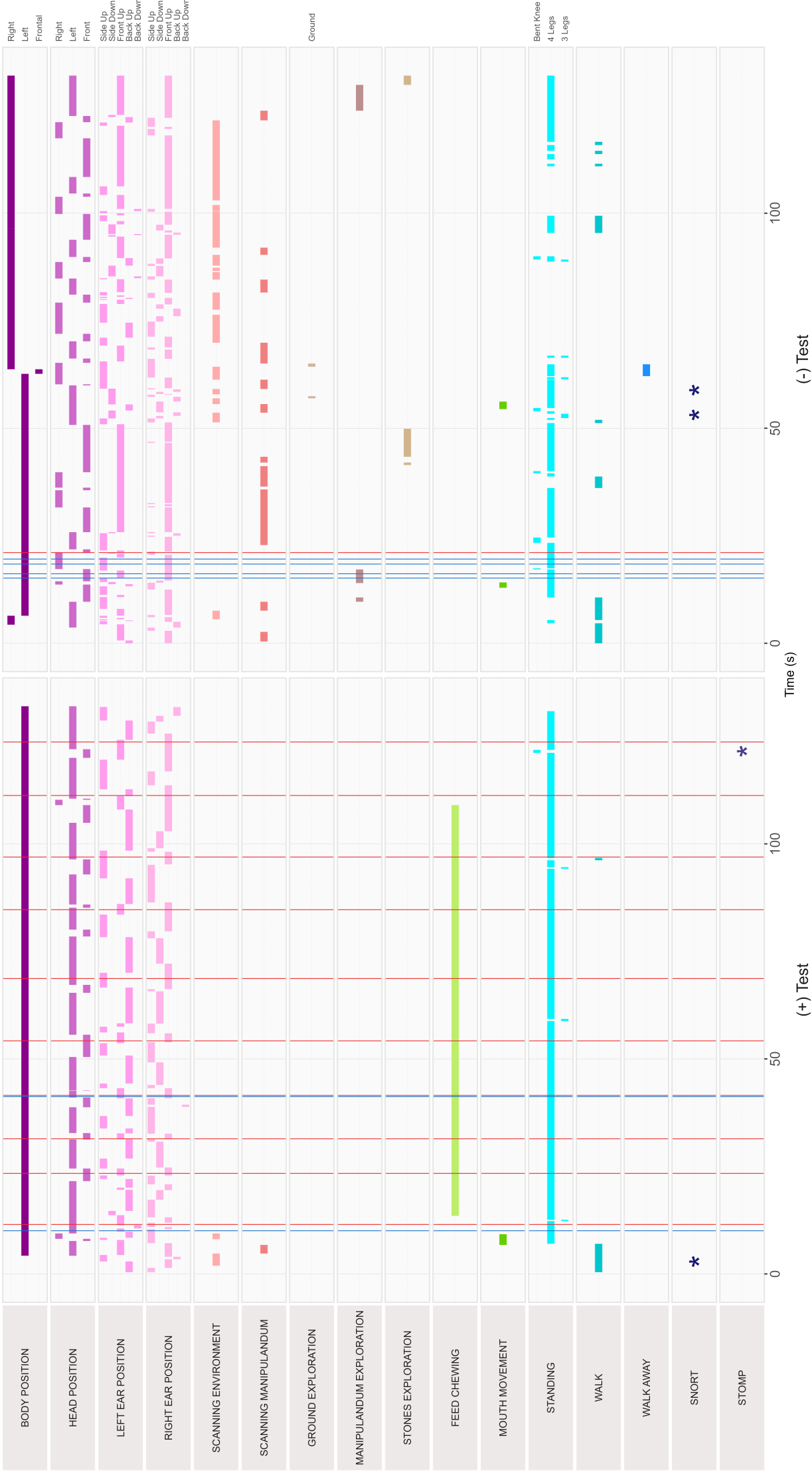

## Marta

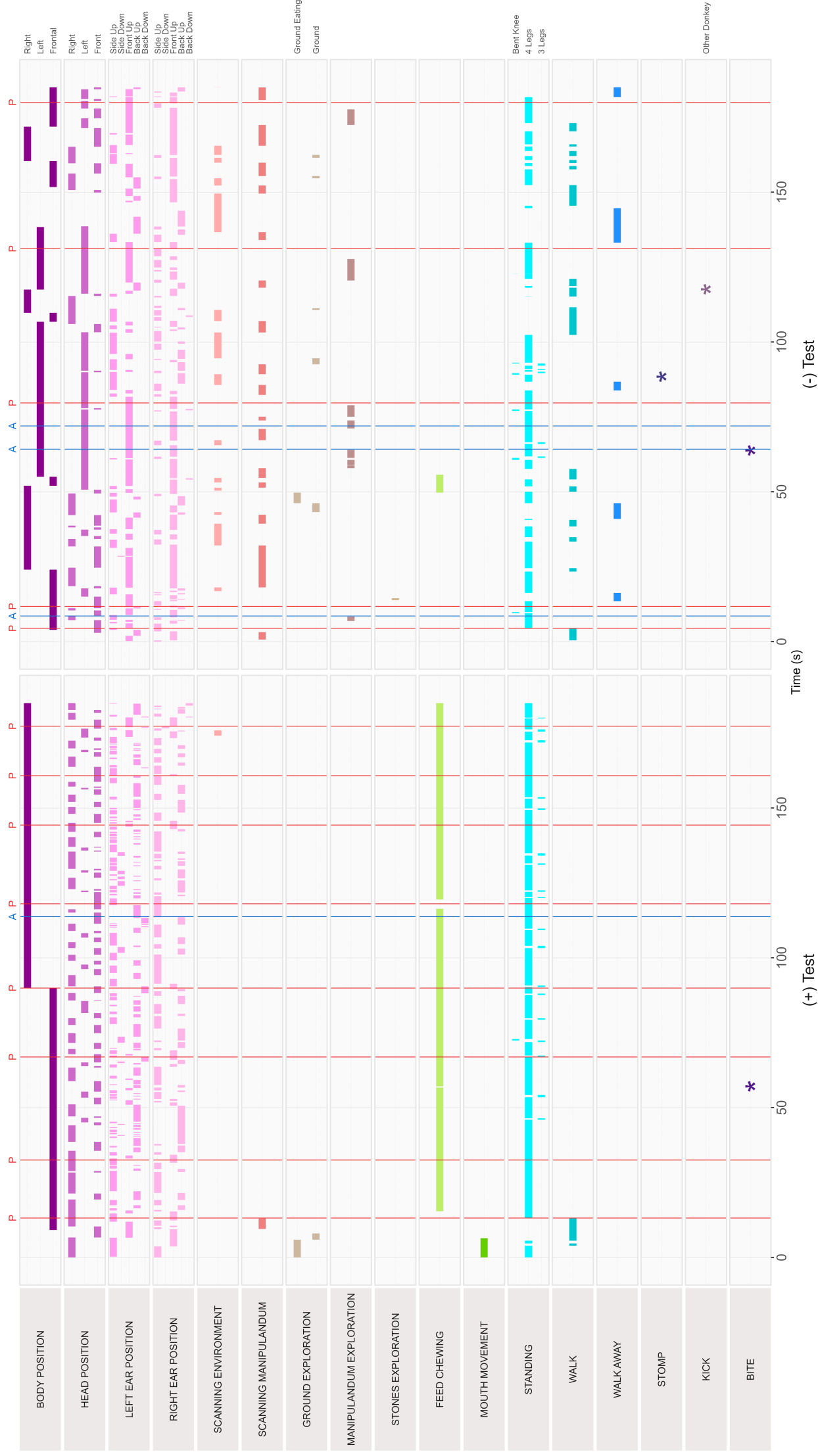

Muchacha

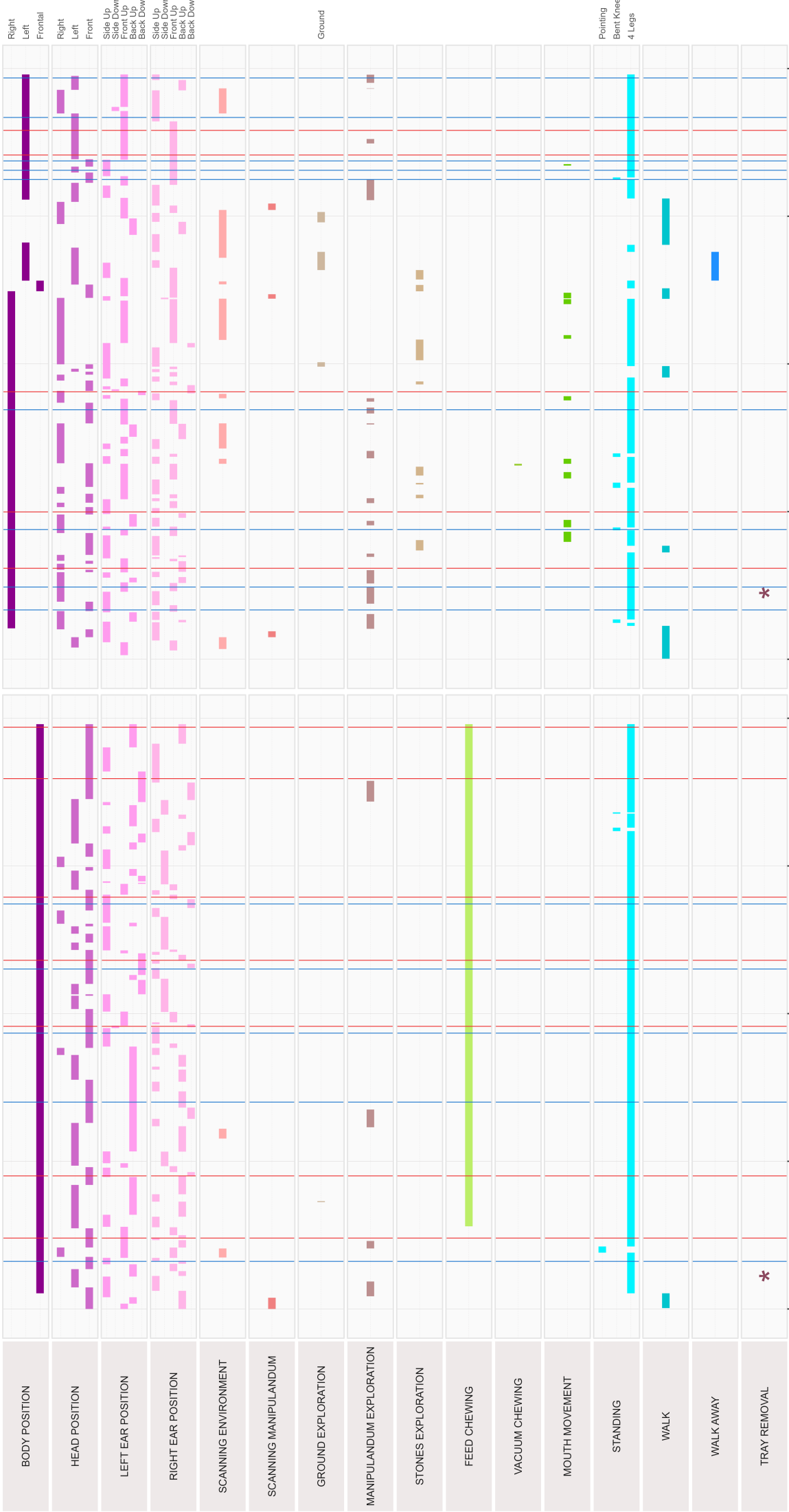

(-) Test

Time (s)

(+) Test

Pepe

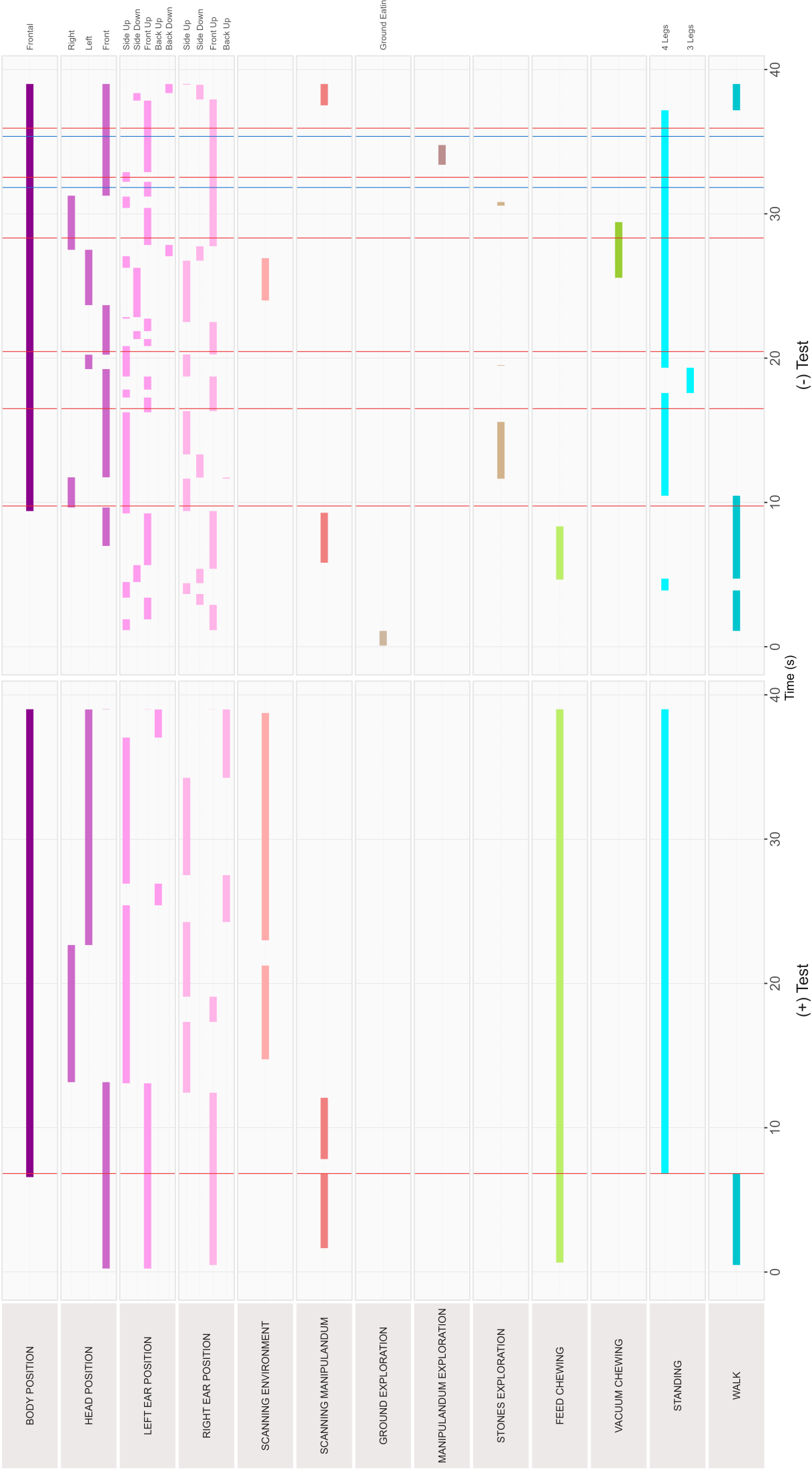

Rosetta

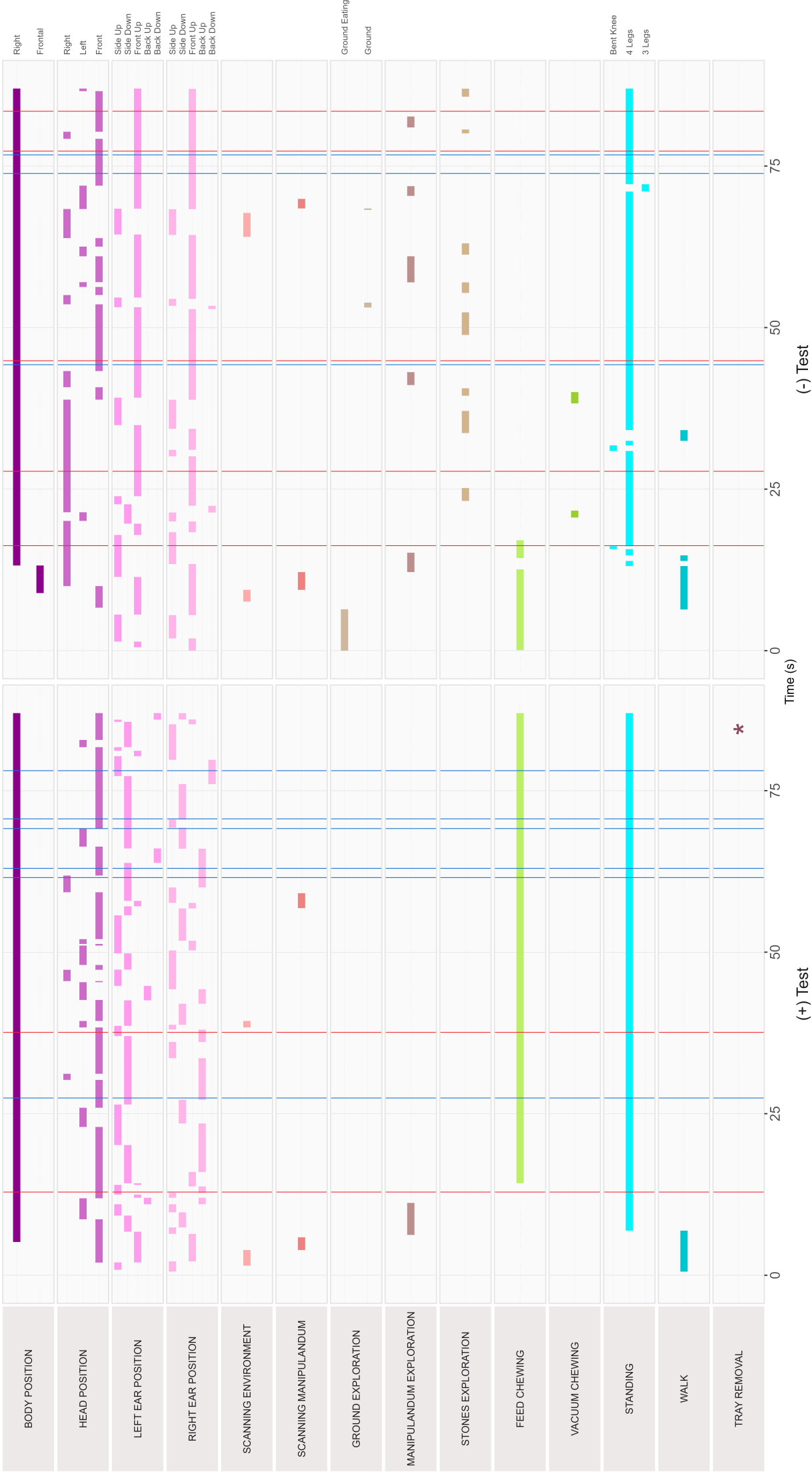

Rosolino

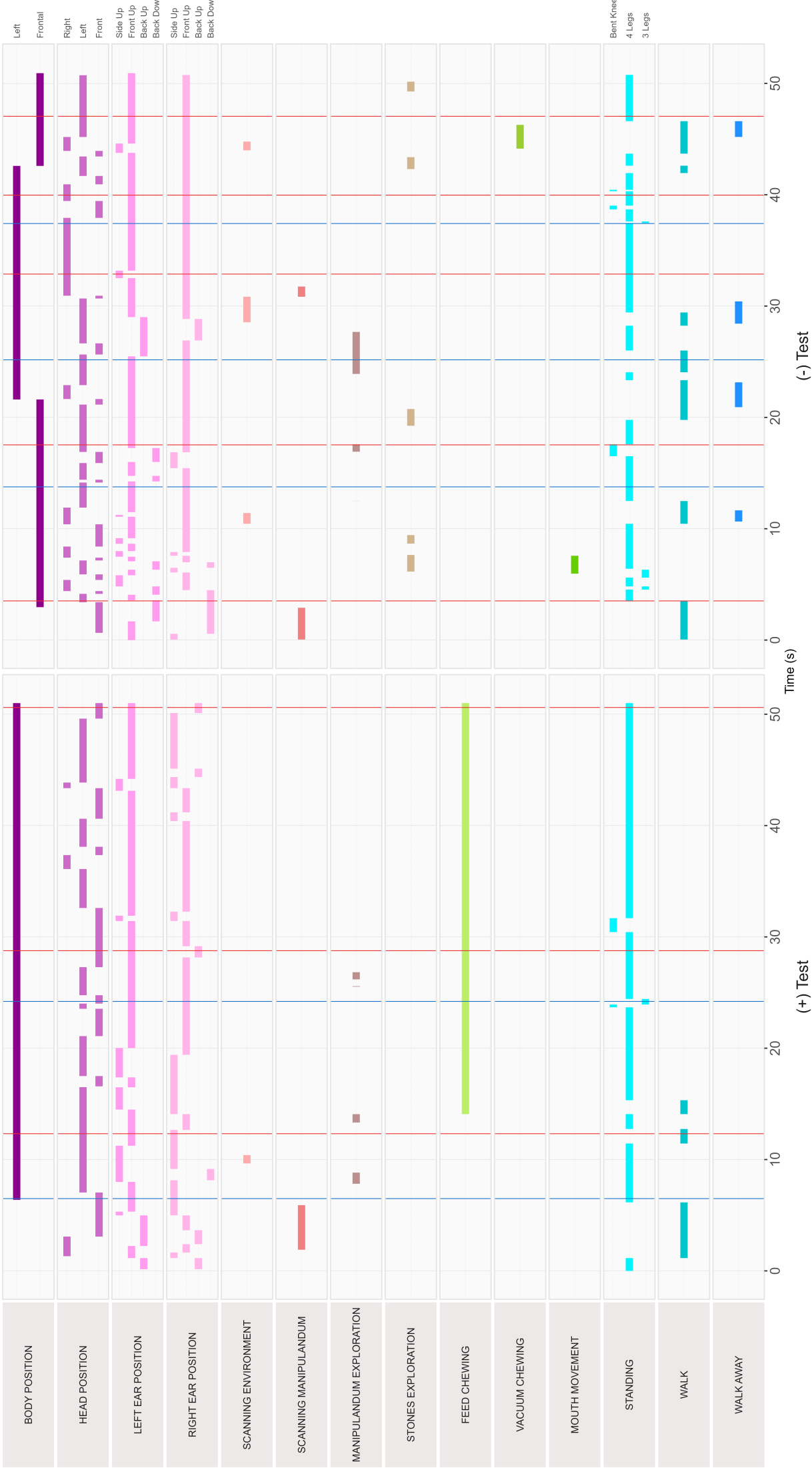

Supplement: Supplementary file 1 [file animals-13-01466-s001.zip › animals-2313160-Figure S1.pdf]
